# Clustering-aided prediction of outcomes in patients with idiopathic pulmonary fibrosis

**DOI:** 10.1186/s12931-024-03015-6

**Published:** 2024-10-23

**Authors:** Lijun Wang, Peitao Wu, Yi Liu, Divya C Patel, Thomas B Leonard, Hongyu Zhao

**Affiliations:** 1 Boehringer Ingelheim Pharmaceuticals, Inc, 900 Ridgebury Road, Ridgefield, CT 06877 USA; 2 Department of Biostatistics, Yale School of Public Health, New Haven, CT USA

**Keywords:** Biomarkers, Disease progression, Interstitial lung disease

## Abstract

**Background:**

Blood biomarkers predictive of the progression of idiopathic pulmonary fibrosis (IPF) would be of value for research and clinical practice. We used data from the IPF-PRO Registry to investigate whether the addition of “omics” data to risk prediction models based on demographic and clinical characteristics improved prediction of the progression of IPF.

**Methods:**

The IPF-PRO Registry enrolled patients with IPF at 46 sites across the US. Patients were followed prospectively. Median follow-up was 27.2 months. Prediction models for disease progression included omics data (proteins and microRNAs [miRNAs]), demographic factors and clinical factors, all assessed at enrollment. Data on proteins and miRNAs were included in the models either as raw values or based on clusters in various combinations. Least absolute shrinkage and selection operator (Lasso) Cox regression was applied for time-to-event composite outcomes and logistic regression with L1 penalty was applied for binary outcomes assessed at 1 year. Model performance was assessed using Harrell’s C-index (for time-to-event outcomes) or area under the curve (for binary outcomes).

**Results:**

Data were analyzed from 231 patients. The models based on demographic and clinical factors, with or without omics data, were the top-performing models for prediction of all the time-to-event outcomes. Relative changes in average C-index after incorporating omics data into models based on demographic and clinical factors ranged from 1.7 to 3.2%. Of the blood biomarkers, surfactant protein-D, serine protease inhibitor A7 and matrix metalloproteinase-9 (MMP-9) were among the top predictors of the outcomes. For the binary outcomes, models based on demographics alone and models based on demographics plus omics data had similar performances. Of the blood biomarkers, CC motif chemokine 11, vascular cell adhesion protein-1, adiponectin, carcinoembryonic antigen and MMP-9 were the most important predictors of the binary outcomes.

**Conclusions:**

We identified circulating protein and miRNA biomarkers associated with the progression of IPF. However, the integration of omics data into prediction models that included demographic and clinical factors did not materially improve the performance of the models.

**Trial registration:**

ClinicalTrials.gov; No: NCT01915511; registered August 5, 2013; URL: www.clinicaltrials.gov.

**Supplementary Information:**

The online version contains supplementary material available at 10.1186/s12931-024-03015-6.

## Background

Idiopathic pulmonary fibrosis (IPF) is a progressive fibrosing interstitial lung disease that leads to loss of lung function and premature death [1]. Although IPF is always progressive, it progresses more quickly in some patients than in others [2, 3]. Several demographic and clinical characteristics have been associated with decline in lung function or risk of mortality in patients with IPF [3–6], but it remains challenging to predict the risk of short-term progression for an individual patient.

The identification of circulating biomarkers predictive of the progression of IPF would be of value for research and clinical practice. “Omics” techniques such as genomics, transcriptomics, proteomics and metabolomics may help to identify molecular biomarkers predictive of disease progression. Clustering allows molecular data to be summarized in an informative way. Although clustering methods cannot be used directly to predict the risk of outcomes, clustering data can be incorporated into risk prediction models to improve their performance [7].

The IPF-PRO Registry is a prospective multicenter US registry of patients with IPF [8]. In a previous analysis of data from this registry, two novel endotypes of IPF were identified by integrating data on circulating proteins and microRNAs (miRNAs) using an unsupervised clustering method [9]. These two endotypes were associated with distinct clinical characteristics and differing risks of disease progression [9]. In a separate analysis of data from US- and UK-based cohorts, clustering analyses based on gene expression profiles from 220 patients identified three endotypes of IPF, which differed in survival [10]. We used data from the IPF-PRO Registry to investigate whether the addition of omics data (circulating proteins and miRNAs) to risk prediction models based on demographic and clinical characteristics improved the prediction of clinically relevant outcomes.

## Methods

### Patients

The IPF-PRO Registry (ClinicalTrials.gov; No: NCT01915511; registered August 5, 2013) enrolled patients with IPF that was diagnosed or confirmed at the enrolling center within the prior six months at 46 centers across the USA. Whole blood and plasma samples were collected at enrollment and stored centrally. Proteins were assayed using a platform encompassing 1305 aptamers (SOMAscan, SOMALogic, Inc). miRNA was isolated using the miRNeasy Serum/Plasma Advanced Kit (Qiagen). Patients were followed prospectively, with data collected as part of routine clinical care until death, lung transplant, or withdrawal from the registry. Follow-up from a centralized call center confirmed patients’ vital status every six months.

The study was conducted in accordance with the Declaration of Helsinki, and it was approved by the Duke University Institutional Review Board (Pro00046131). The protocol was approved by the relevant Institutional Review Boards and/or local Independent Ethics Committees at each site. All patients provided written informed consent.

### Prediction models

Our prediction models included omics data (44 proteins and 472 miRNAs), demographic factors and clinical factors, all assessed at enrollment into the registry. The demographic factors were age, sex, body mass index (BMI), smoking status (ever vs. never), treatment with nintedanib and treatment with pirfenidone. The clinical factors were per cent predicted values for forced vital capacity (FVC) and diffusing capacity of the lungs for carbon monoxide (DLco). Base models included only demographic and/or clinical variables. Data on proteins and miRNAs were included in the models either as the raw values or based on the cluster label (indicating the group membership of each patient when the patients were grouped into clusters using proteins or miRNAs) in various combinations. Since data on proteins and miRNAs might produce different clusters, we used the individual clustering results from different omics and incorporated them into a generalized linear model. The models are described in more detail in Additional file 1.

The time to event outcomes studied were composite outcomes including death (Table 1). In addition, two binary outcomes were assessed: absolute decline in FVC % predicted > 10% at 1 year and disease progression (absolute decline in FVC % predicted > 10%, absolute decline in DLco % predicted > 15%, death, or lung transplant) at 1 year.

**Table 1:** Proportions of patients with composite outcomes (*N* = 231)

Death or lung transplant	Death, lung transplant, or decline in FVC ≥ 10%	Death, lung transplant, or decline in DLco % predicted ≥ 15%	Death, lung transplant, or respiratory hospitalization
95 (41%)	142 (62%)	116 (50%)	109 (47%)

Data are n(%). The median follow-up time was 27.2 months. Abbreviations: FVC: forced vital capacity, DLco: diffusing capacity of the lungs for carbon monoxide

### Workflow

**Fig. 1:**
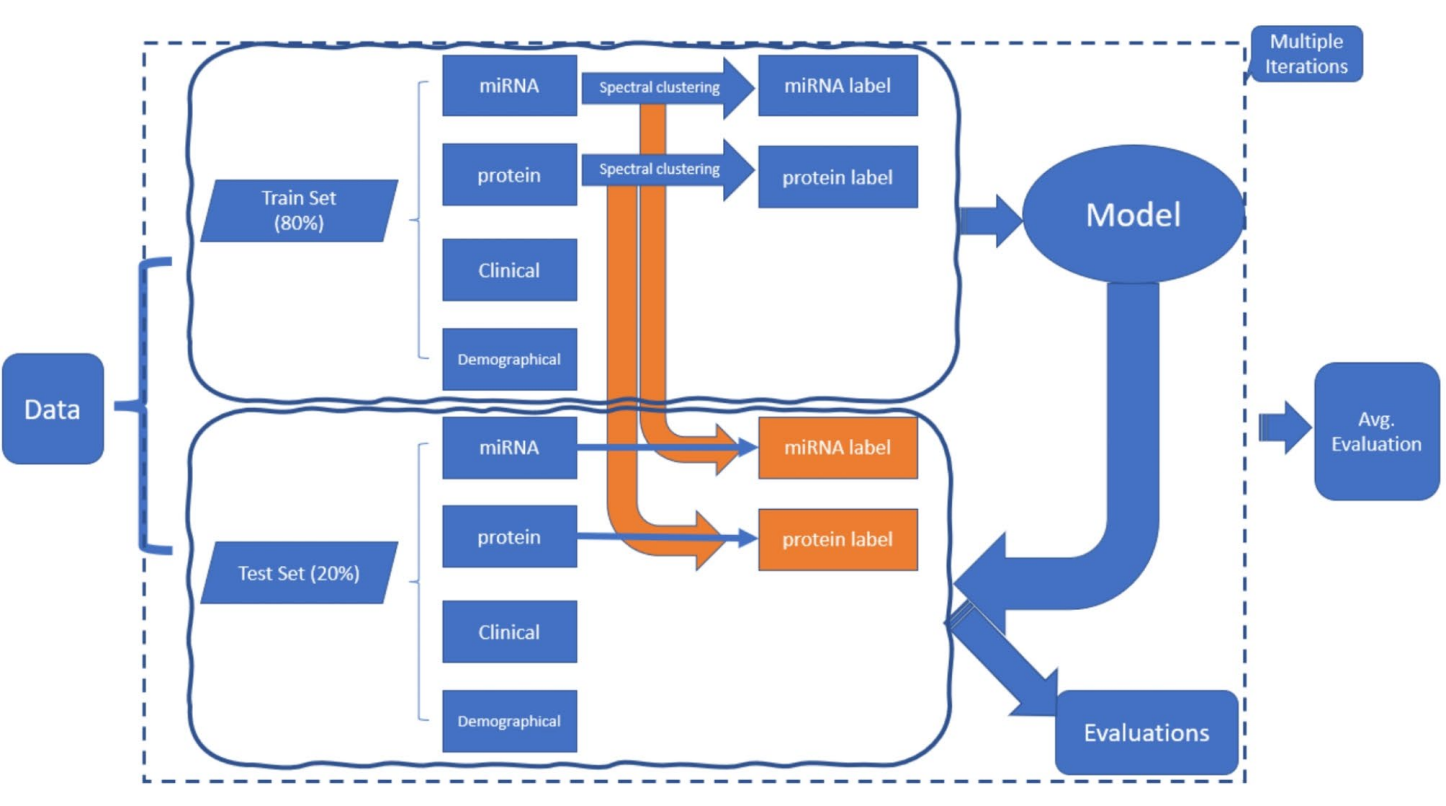
Workflow for evaluating the performance of different models

We used the following workflow to compare the predictive performance of different models (Fig. 1). The data were split into a training set (80%) and a test set (20%). Spectral clustering was performed on the protein and miRNA data in the training set to make predictions for the test set. Least absolute shrinkage and selection operator (Lasso) Cox regression was applied for time-to-event data and logistic regression with L1 penalty was applied for the binary outcomes. The cluster label for the test set was determined and the performance of models that included the cluster label were assessed using Harrell’s C-index for time-to-event outcomes and area under the curve (AUC) for binary outcomes. A 10-fold cross-validation procedure was performed. Randomness came from splitting the data into the training and test sets and from model tuning. The workflow was repeated multiple times, and the average C-index for the Cox model for time-to-event data, or AUC for binary responses, was used to estimate final model performance. An average C-index or AUC > 0.5 indicated that model-based prediction was better than a random guess. Variables were ranked by their importance as predictors of the outcomes.

Relative per cent changes in the average C-index before versus after incorporating the omics data into models based only on demographics, or on demographic plus clinical factors, were calculated to determine the degree of improvement in risk prediction. For these calculations, the omics data incorporated were raw protein values and the cluster label of the miRNAs.

## Results

### Time to event outcomes

**Fig. 2:**
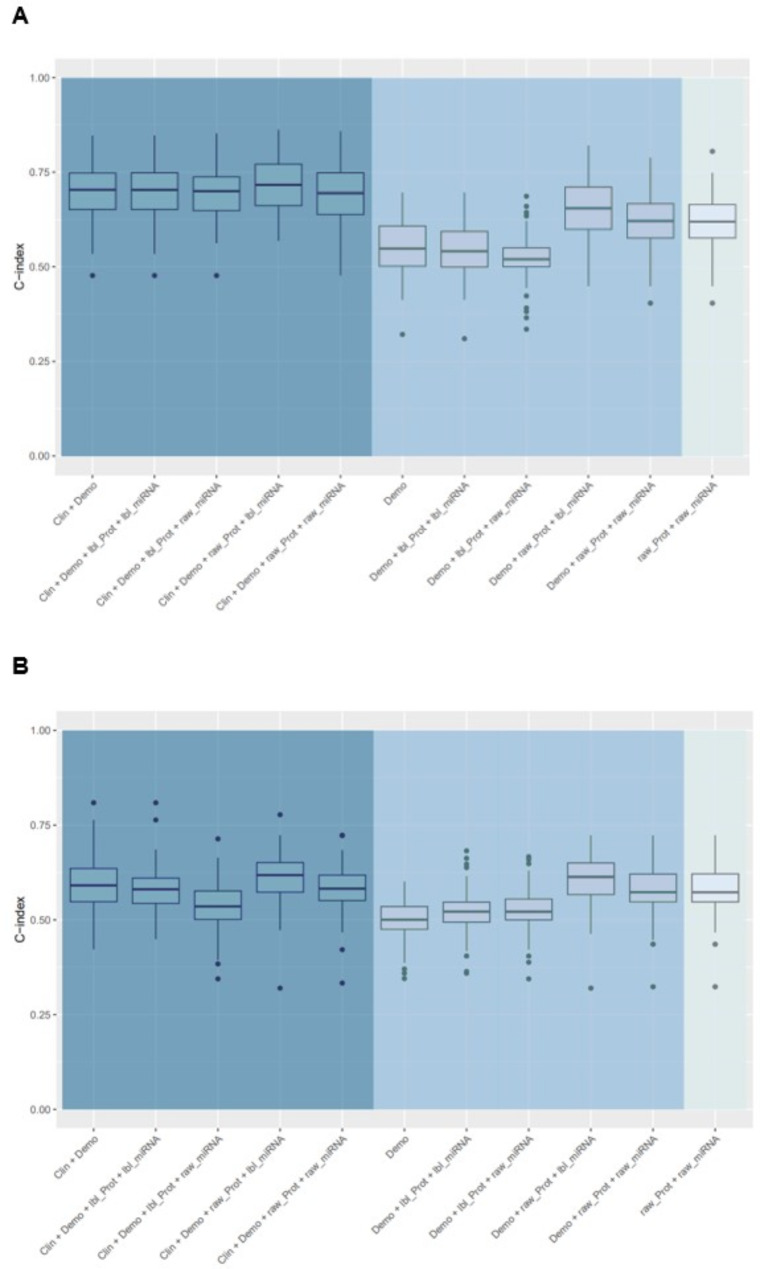
C-indices of models for (**A**) composite of time to death or lung transplant and (**B**) composite of time to death, lung transplant, or decline in forced vital capacity > 10%. The left panel shows models based on demographic and clinical characteristics with or without omics data. The middle panel shows models based on demographics with or without omics data. The right panel shows the model based on omics data alone. Clin, clinical; demo, demographics; lbl_miRNA, cluster label of miRNA; lbl_prot, cluster label of protein; raw_miRNA, raw values of miRNAs; raw_prot, raw values of proteins.

Demographic, clinical and omics data were merged from 231 patients. The baseline characteristics of these patients are summarized in Table 2. The median follow-up was 27.2 months. Overall, 64 repetitive experiments were conducted to investigate the performance of the candidate models. The proportion of patients who experienced the composite outcomes are shown in Table 1. C-indices for each model for the composite of death or lung transplant and the composite of death, lung transplant, or decline in FVC % predicted > 10% are shown in Fig. 2A and B. C-indices for these outcomes from models that also included oxygen use at rest are shown in Fig S1 and S2. C-indices for each model for the other composite outcomes are shown in Fig.S3 and Fig. S4 in Additional file 1. For all the composite outcomes, the model based on the raw values of the proteins and the raw values of miRNAs alone yielded a C-index greater than 0.5. However, for all the composite outcomes, the models based on demographic and clinical factors, with or without the integration of omics data, were the top-performing models. The relative percent change in the average C-index after incorporating omics information into the prediction models based on demographic factors ranged from 12.4 to 21.2% (Table 3). The relative percent change in the average C-index after incorporating omics information into the prediction models based on demographic and clinical factors (FVC % predicted and DLco % predicted) ranged from 1.7 to 3.2% (Table 3). Correlations between FVC % predicted and DLco % predicted and the proteins were generally weak (Fig. S5).

**Table 2:** Characteristics of analysis cohort at enrollment into IPF-PRO Registry (*N* = 231)

Male	171 (74.0)
Age, years	69.7 (7.8)
Body mass index*	29.4 (4.8)
Ever smoker	155 (67.1)
FVC % predicted	71.1 (16.9)
DLco % predicted^†^	40.2 (13.6)
Taking nintedanib	42 (18.2)
Taking pirfenidone	83 (35.9)

Data are No. (%) or mean (SD). *Data were not available for 6 patients. ^†^Data were not available for 59 patients. Abbreviations: FVC: forced vital capacity, DLco: diffusing capacity of the lungs for carbon monoxide

**Table 3:** Average C-indices of models before and after incorporating omics information

	Death or lung transplant	Death, lung transplant or decline in FVC ≥ 10%	Death, lung transplant or decline in DLco % predicted ≥ 15%	Death, lung transplant or respiratory hospitalization
**Demographics**				
Demographics	0.552 (0.009)	0.497 (0.007)	0.532 (0.008)	0.563 (0.008)
Demographics plus raw values of proteins and cluster label of miRNA	0.669 (0.009)	0.592 (0.010)	0.636 (0.009)	0.632 (0.008)
Percent change after integration of omics information	21.2	19.0	19.5	12.4
**Demographic and clinical factors**				
Demographic and clinical factors	0.700 (0.009)	0.594 (0.009)	0.644 (0.009)	0.681 (0.008)
Demographics plus clinical factors plus raw values of proteins and cluster label of miRNA	0.722 (0.008)	0.606 (0.009)	0.663 (0.008)	0.692 (0.008)
Percent change after integration of omics information (%)	3.2	1.9	2.8	1.7

Data are mean (SE) unless otherwise stated. Abbreviations: FVC: forced vital capacity, DLco: diffusing capacity of the lungs for carbon monoxide

**Fig. 3:**
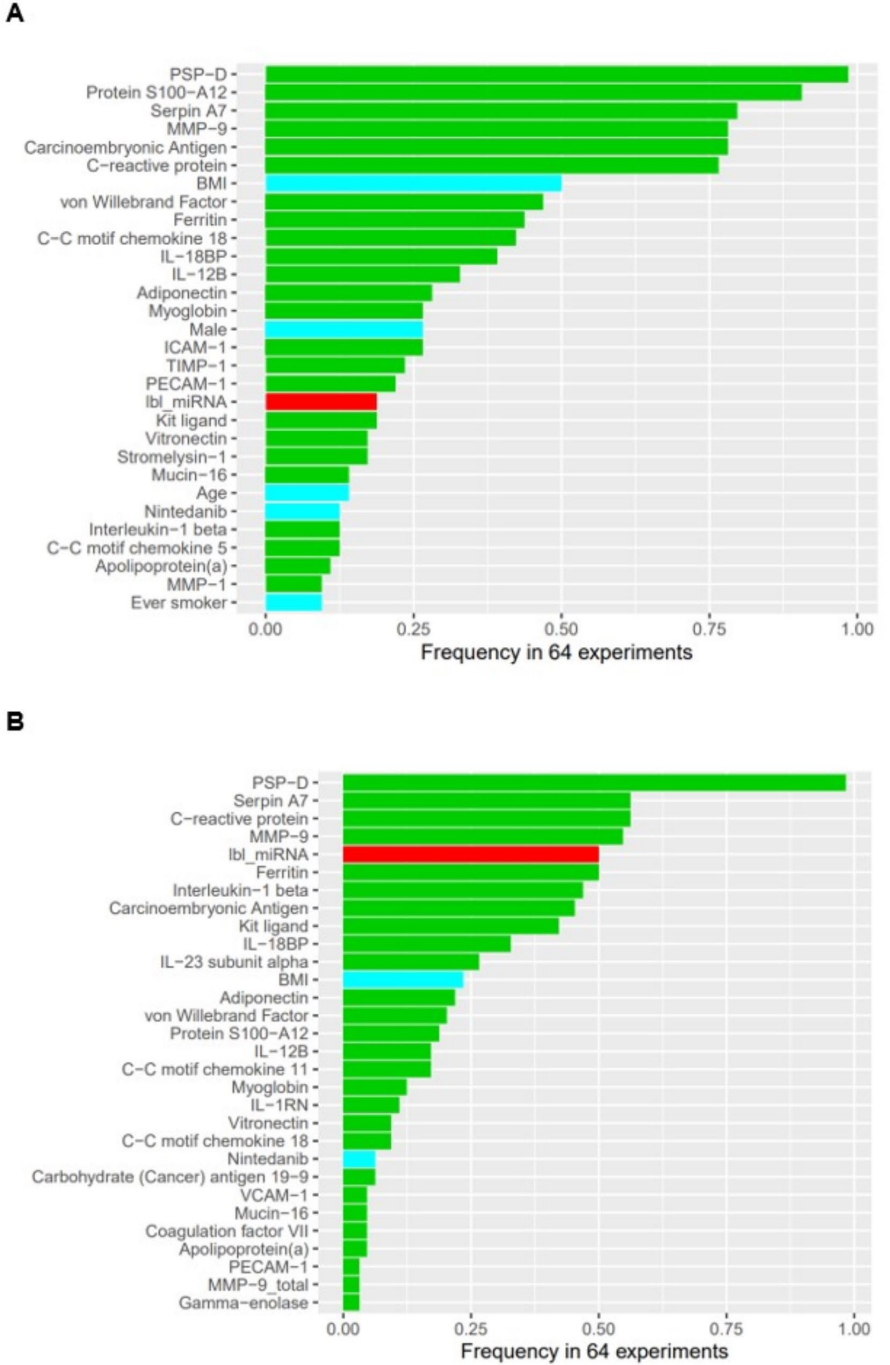
Variable importance of demographic and omics variables for (**A**) composite outcome of death or lung transplant and (**B**) composite outcome of death, lung transplant, or decline in forced vital capacity (FVC) > 10%. Green bars denote proteins, blue bars denote demographic variables and the red bar denotes the cluster label of miRNA. Demo, demographics; lbl_miRNA, clustering label of miRNA; raw_prot, raw values of proteins

The importance of variables in the model including demographics, raw protein values and the cluster label of miRNAs are summarized in Fig. 3A and B for the composite outcome of death or lung transplant and the composite outcome of death, lung transplant, or decline in FVC > 10% and in Fig. S6 and Fig. S7 in Additional file 1 for the other outcomes. Surfactant protein-D (PSP-D) was the most important predictor of these composite outcomes. Serine protease inhibitor (serpin) A7 and matrix metalloproteinase-9 (MMP-9) were among the top predictors of each outcome.

Re-fitting the models using the top four protein predictors plus the clinical and demographic factors and omics data for the composite of death or lung transplant and the composite of death, lung transplant, or decline in FVC % predicted > 10% improved the performance of the models compared with the Lasso models (Fig. S8 and Fig. S9).

### Binary outcomes

**Fig. 4:**
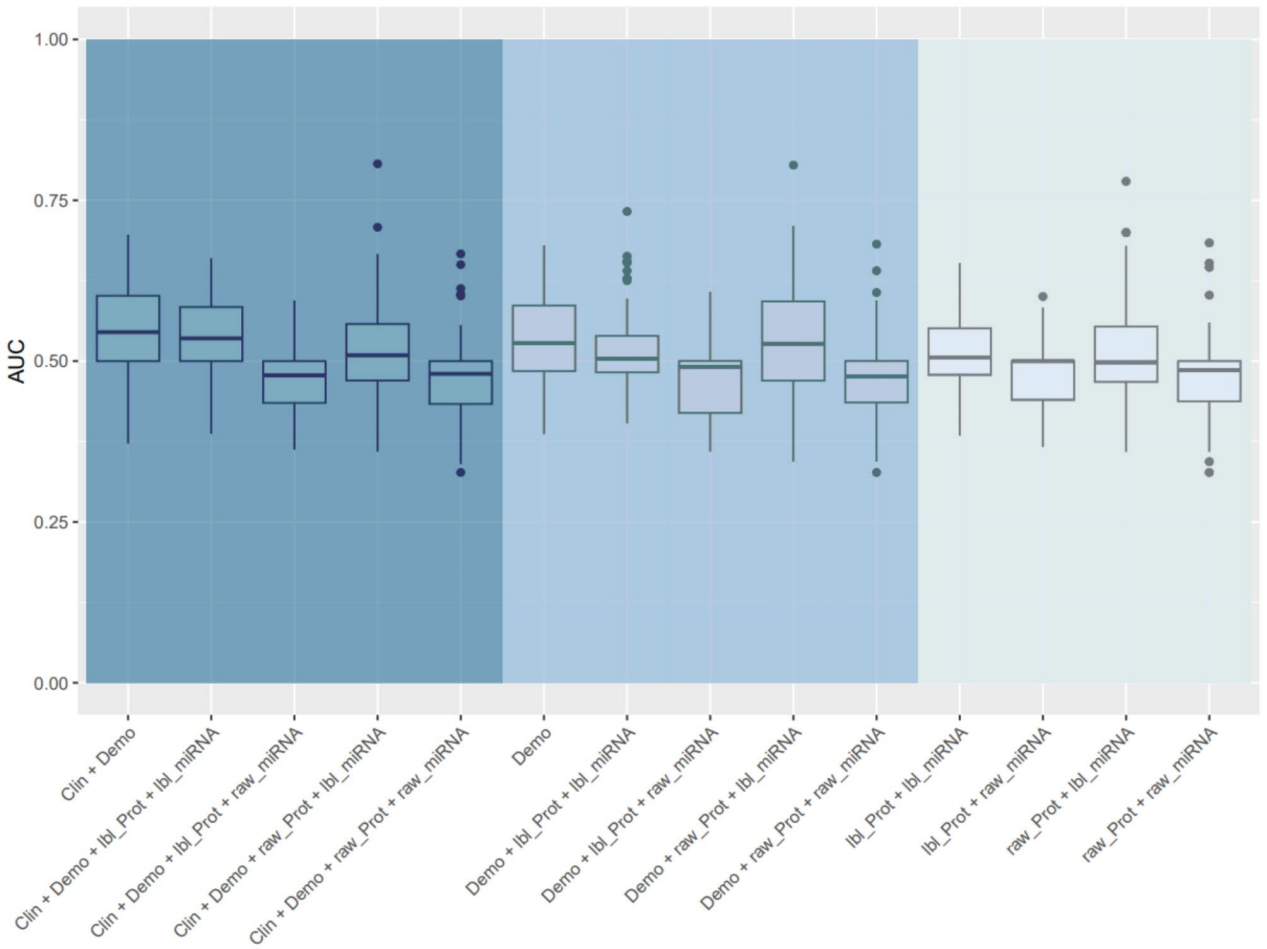
Area under the curve (AUC) of models for absolute decline in forced vital capacity > 10% at 1 year. The left panel shows models based on demographic and clinical characteristics with or without omics data. The middle panel shows models based on demographics with or without omics data. The right panel shows the models based on omics data alone. Clin, clinical; demo, demographics; lbl_miRNA, cluster label of miRNA; lbl_prot, cluster label of protein; raw_miRNA, raw values of miRNAs; raw_prot, raw values of proteins

**Fig. 5:**
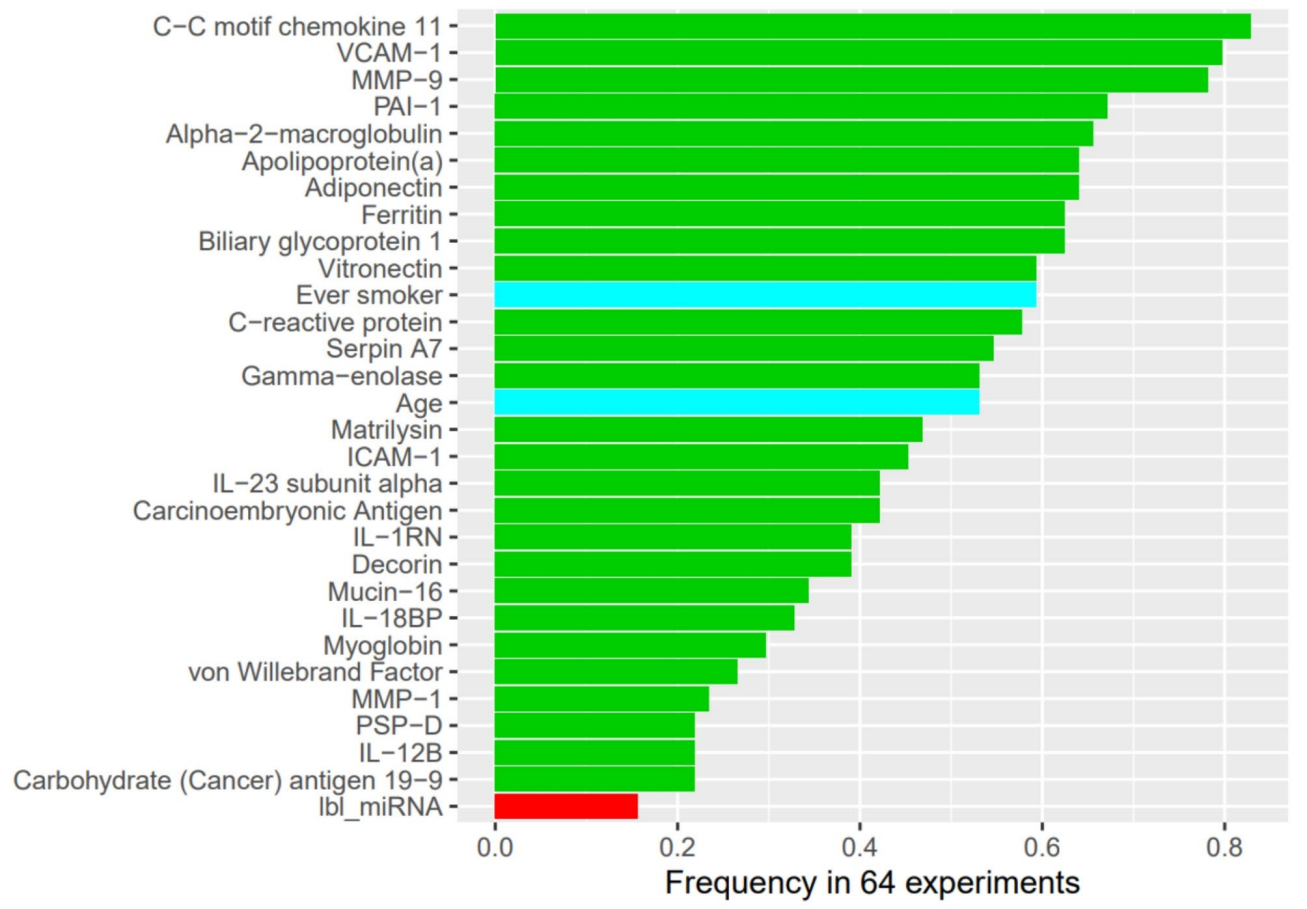
Variable importance of demographic and omics variables for absolute decline in forced vital capacity > 10% at 1 year. Green bars denote proteins, blue bars denote demographic variables and the red bar denotes the cluster label of miRNA. Demo, demographics; lbl_miRNA, clustering label of miRNA; raw_prot, raw values of proteins

One patient had missing data for the binary outcome of absolute decline in FVC % predicted > 10% so the analysis cohort for this outcome was 230 patients. The proportions of patients with an absolute decline in FVC > 10% at 1 year and disease progression at 1 year were 42.0% and 48.3%. For the outcome of absolute decline in FVC > 10% at 1 year, the model based on demographics alone and the model based on demographic and omics data had a similar performance, as did the model based on demographic and clinical factors alone and the model based on these factors and omics data (Fig. 4). The importance of each variable in the model for prediction of this outcome based on demographics, raw protein values and the cluster label of miRNA is shown in Fig. 5. CC motif chemokine 11, vascular cell adhesion protein-1 (VCAM-1) and MMP-9 were the most important predictors in this model.

**Fig. 6:**
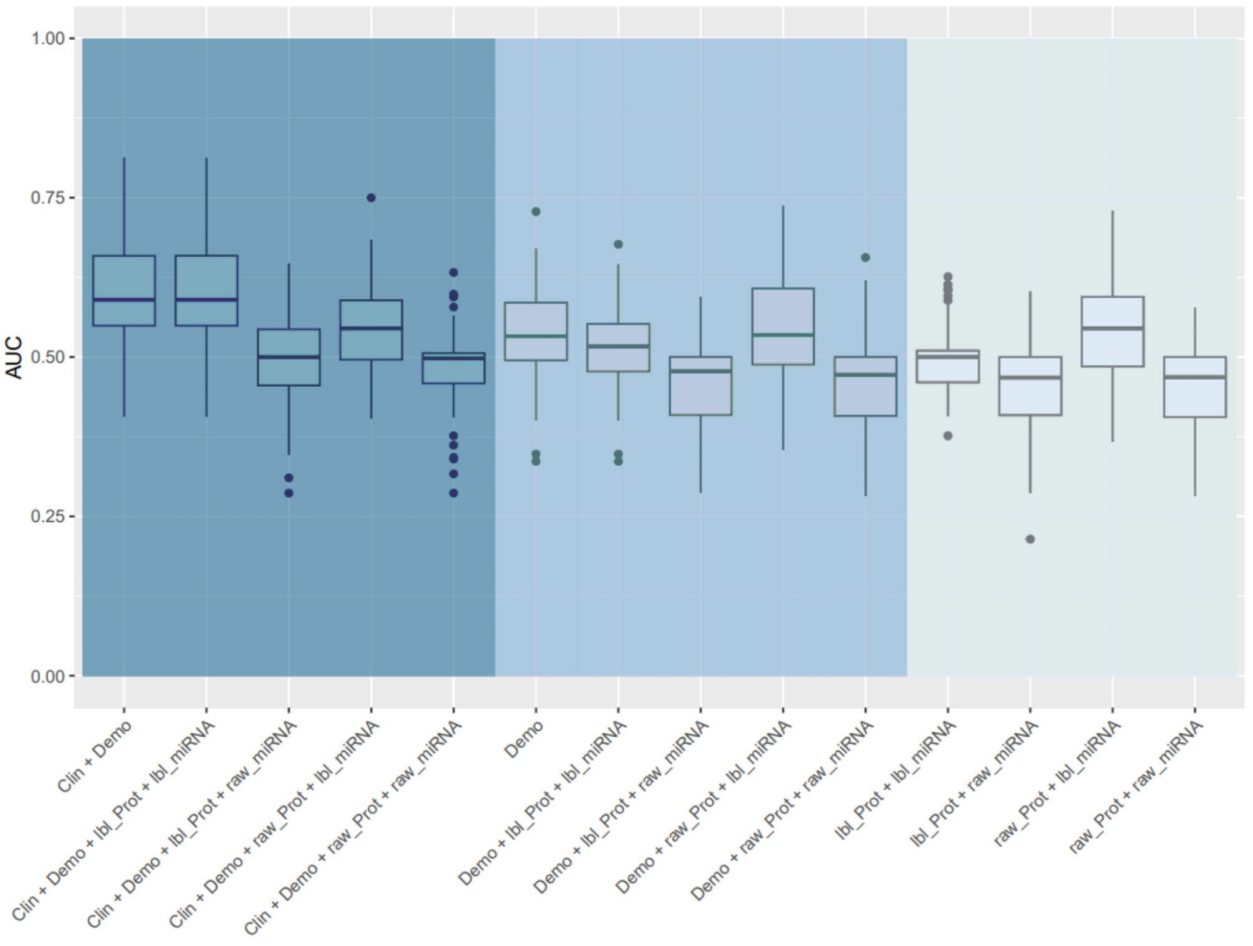
Area under the curve (AUC) of models for disease progression (decline in forced vital capacity [FVC] % predicted > 10%, decline in diffusing capacity of the lungs for carbon monoxide [DLco] % predicted > 15%, death, or lung transplant) at 1 year. The left panel shows models based on demographics and clinical characteristics with or without omics data. The middle panel shows models based on demographics with or without omics data. The right panel shows the models based on omics data alone. Clin, clinical; demo, demographics; lbl_miRNA, cluster label of miRNA; lbl_prot, cluster label of protein; raw_miRNA, raw values of miRNAs; raw_prot, raw values of proteins

**Fig. 7:**
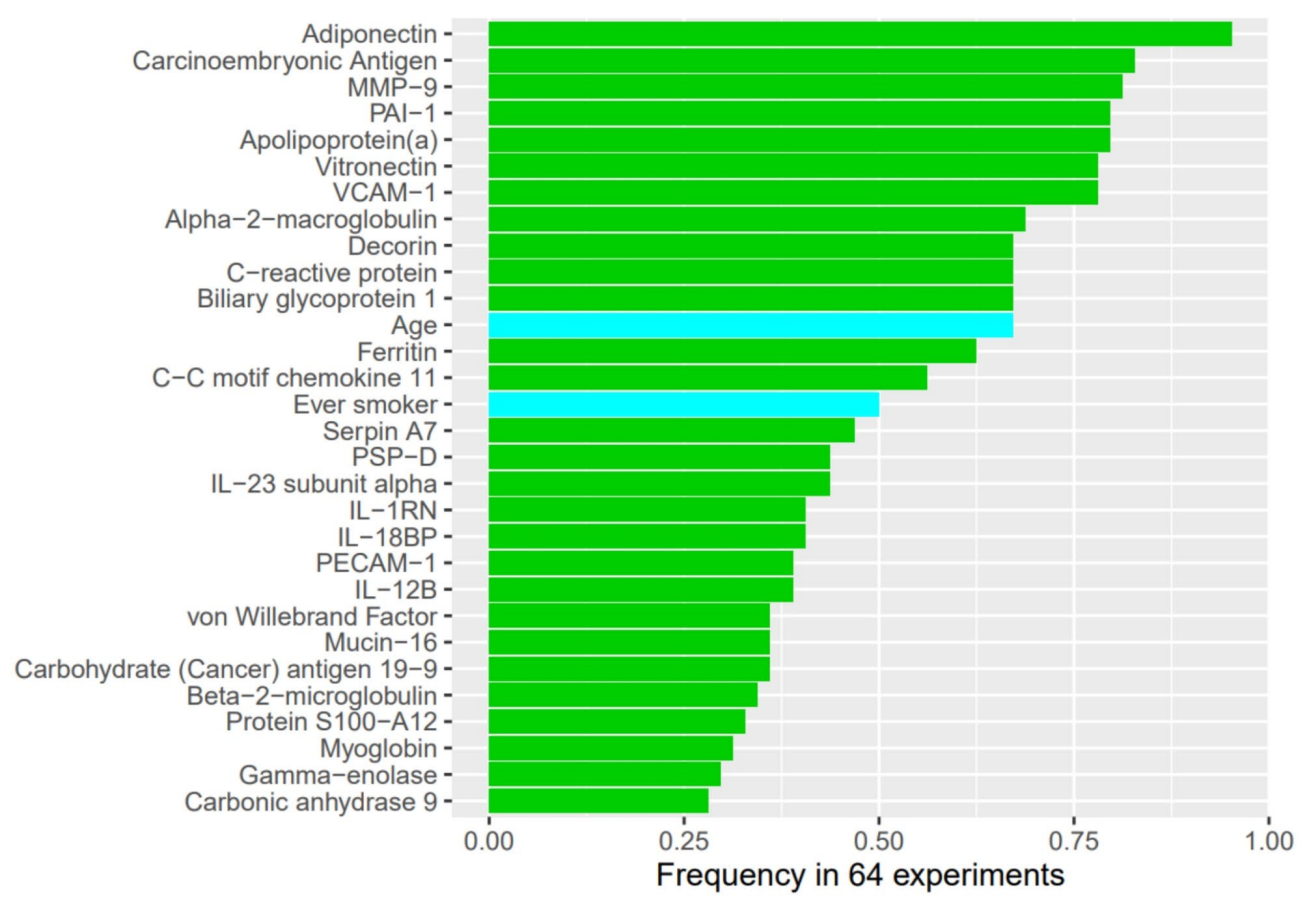
Variable importance by selected frequency for disease progression (decline in forced vital capacity [FVC] % predicted > 10%, decline in diffusing capacity of the lungs for carbon monoxide [DLco] % predicted > 15%, death or lung transplant) at 1 year. Green bars denote proteins, blue bars denote demographic variables and the red bar denotes the cluster label of miRNA. Demo, demographics; lbl_miRNA, clustering label of miRNA; raw_prot, raw values of proteins

For the outcome of disease progression at 1 year, the models based on demographic and clinical factors, with or without the integration of omics data, were the top performing models (Fig. 6). The model based on demographics alone and the model based on demographics plus omics data had a similar performance. The importance of each variable in the model for prediction of this outcome based on demographics, raw protein values and the cluster label of miRNA is shown in Fig. 7. Adiponectin, carcinoembryonic antigen and MMP-9 were the most important predictors in this model.

Re-fitting the models using the top four protein predictors plus the clinical and demographic factors and omics data for the outcomes of absolute decline in FVC % predicted > 10% at 1 year and disease progression at 1 year improved the performance of the models compared with the Lasso models (Fig. S10 and Fig. S11).

## Discussion

We used a clustering-based approach to assess whether the integration of data on circulating proteins and miRNAs improved models based on demographic and clinical factors for the prediction of clinically relevant outcomes in patients with IPF. Our results showed that the addition of omics data resulted in only small improvements to the models based on demographic and clinical factors. This likely reflects the fact that clinical measures such as FVC and DLco % predicted are good measures of disease severity in patients with IPF and are strongly associated with prognosis [6, 11–13]; thus it would be challenging for a circulating biomarker to improve prediction models based on these factors. Previous studies have suggested that a combination of biomarkers and clinical factors may better identify patients with IPF at risk of short-term progression than clinical factors alone [14–17]. In a study of data from 1226 patients, a machine learning-derived model that included proteomic data, FVC % predicted and DLco % predicted outperformed a model based only on sex, FVC % predicted and DLco % predicted in predicting survival over 36 months [17]. A model that combined the GAP score (based on age, sex and lung function) with a progression index based on four serum protein biomarkers selected using LASSO increased the ability to predict progression over one year compared with the GAP score alone [16]. However, other studies have not found that the addition of biomarker data improved outcome prediction compared with models based on clinical factors alone [18] and the utility of circulating biomarkers to improve the prediction of short-term outcomes in clinical practice is yet to be established. No biomarker has consistently been found to perform better than the standard practice of predicting and monitoring progression using clinical factors.

In our study, the biomarkers of greatest importance for prediction of the clinical outcomes included SP-D, plasminogen activator inhibitor 1 (PAI-1), serpin A7, VCAM-1 and MMP-9. Previous studies have also found these biomarkers to be associated with the progression of IPF. Circulating SP-D is a marker of epithelial injury that has been associated with disease progression and mortality in patients with IPF in several studies [19–21]. PAI-1 (also known as serpin 1) is a marker of coagulation and has been observed at higher levels in patients with acute exacerbations of IPF compared with those who were not experiencing a rapid decline in respiratory function [22]. Serpin A7 was identified as a predictor of respiratory death or lung transplant in multivariable analyses of data from the IPF-PRO Registry that considered protein biomarkers with or without clinical variables [23]. VCAM-1 is a marker of inflammation and high levels of VCAM-1 in peripheral blood have been associated with mortality in patients with IPF [24, 25]. Matrix metalloproteinases are involved in extracellular matrix remodeling and angiogenesis [26]. Elevated levels of MMP-9 have been observed in the bronchoalveolar lavage fluid of patients with IPF who had rapid decline in lung function [27] or who died [28]. In our study, the time to event analyses and the analyses of binary FVC outcomes identified different biomarkers as being of greatest importance as predictors of disease progression. This may be because while binary FVC outcomes are commonly used to assess disease progression or response to treatment in clinical studies, they are less likely than time to event analyses to identify biomarkers that play a more time-sensitive role in disease progression. Further research is needed into which biomarkers are of greatest value as predictors or markers of the progression of IPF.

Strengths of our study include the prospective follow-up of patients in the IPF-PRO Registry and the central testing of the blood biomarkers. Limitations include the use of an aptamer-based platform for measuring circulating proteins, rather than an unbiased approach, and the variable disease severities of the patients enrolled in the registry, as it may be that different biomarkers are important predictors of the progression of IPF in patients in different phases of the disease. Our model only captured linear effects, with variables selected via Lasso, and there might be interaction effects across biomarkers and other features. The sample size of 231 patients may have provided inadequate power to detect effects of certain biomarkers.

## Conclusions

These analyses of data from the IPF-PRO Registry identified protein and miRNA biomarkers associated with the progression of IPF. However, the integration of data on protein and miRNA biomarkers into prediction models that included demographic and clinical factors did not materially improve the performance of the models. Further research is needed to inform the clinical utility of circulating biomarkers in patients with IPF.

## Electronic supplementary material

Below is the link to the electronic supplementary material.

Supplementary Material 1

## Data Availability

The datasets analyzed during the current study are not publicly available, but are available from the corresponding author on reasonable request.
