# Supplementary material for: Clustering-aided prediction of outcomes in patients with idiopathic pulmonary fibrosis
Source: Respir Res. 2024 Oct 23;25:383. doi: 10.1186/s12931-024-03015-6 (PMC11515489; doi:10.1186/s12931-024-03015-6)

Clustering-aided prediction of outcomes in patients with idiopathic pulmonary fibrosis

**Additional file 1**

**Statistical methods**

We analyzed clinical factors, demographic factors and omics data (proteins and miRNAs), all assessed at enrollment.

Let $C$ be clinical factors:

- Forced vital capacity (FVC) % predicted
- Diffusing capacity of the lungs for carbon monoxide (DLco) % predicted

Let $D$ be demographic factors:

- Age
- Sex
- Body mass index (BMI)
- Smoking status (ever vs never)
- Treatment with nintedanib (yes/no)
- Treatment with pirfenidone (yes/no)

Consider *base models* that are only based on clinical or demographic factors.

$$M_{Clin}:g\left( Y \right)=C\gamma_{C}$$

$$M_{Demo}:g\left( Y \right)=D\gamma_{D}$$

$$M_{Clin+Demo}:g\left( Y \right)=C\gamma_{C}+D\gamma_{D}$$

If $Y$ is a time-to-event variable, then $g\left( Y \right)=log\frac{h(t)}{h_{0}\left( t \right)}$ is the log hazard ratio in Cox model. If $Y\in\{0, 1\}$ is the binary FVC variable, $g\left( Y \right)= log\frac{P(Y=1)}{1-P(Y=1)}$ is the logit linking function.

Let $X_{i}\in R^{p_{i}}, i=1, 2$ be the measurement of $p_{1}$ proteins and $p_{2}$ miRNA, respectively.

Consider the class of models:

$$M_{Z+f}:g(Y)=f\left( X_{1},X_{2} \right)+Z\gamma,$$

where $Z\in\{Clin,Demo,Clin+Demo\}$ and $Clin+Demo$ means the combination of clinical and demographic factors. An example of $f$ is $f\left( X_{1},X_{2} \right)=X_{1}\beta_{1}+X_{2}\beta_{2}$.

Thus, the question *“Can omics information (proteins and miRNAs) improve risk prediction?”* can be formulated as *“Would* $M_{Z+f}$ *be better than* $M_{Z}$*, where* $Z\in\{Clin, Demo, Clin+Demo\}$*?”* We incorporated the clustering label information into the prediction model, which was expected to summarize information from many variables with weak influence and avoid overfitting by dimension reduction.

Let $L_{i}\in\{1,\ldots,k\},i=1,2$ be the associated cluster labels for $X_{i}$, where $k$ is the number of clusters. Then, we considered the following candidate models,

$$M_{Z+raw\_Prot+raw\_miRNA}:f\left( X_{1},X_{2} \right)=X_{1}\beta_{1}+X_{2}\beta_{2}$$

$$M_{Z+raw\_Prot+lbl\_miRNA}:f\left( X_{1},X_{2} \right)=X_{1}\beta_{1}+L_{2}\beta_{2}$$

$$M_{Z+lbl\_Prot+raw\_miRNA}:f\left( X_{1},X_{2} \right)=L_{1}\beta_{1}+X_{2}\beta_{2}$$

$$M_{Z+lbl\_Prot+lbl\_miRNA}:f\left( X_{1},X_{2} \right)=L_{1}\beta_{1}+L_{2}\beta_{2}$$

where *raw_Prot, lbl_Prot* denoted the raw value and clustering label for protein, respectively, similar for *raw_miRNA* and *lbl_miRNA*. Since $L_{i}$ is the summarized information from $X_{i}$, there was no need to consider the combinations $X_{i}+L_{i}$.

**Fig. S1** C-indices of models including and not including supplemental oxygen use at rest for the composite of time to death or lung transplant.


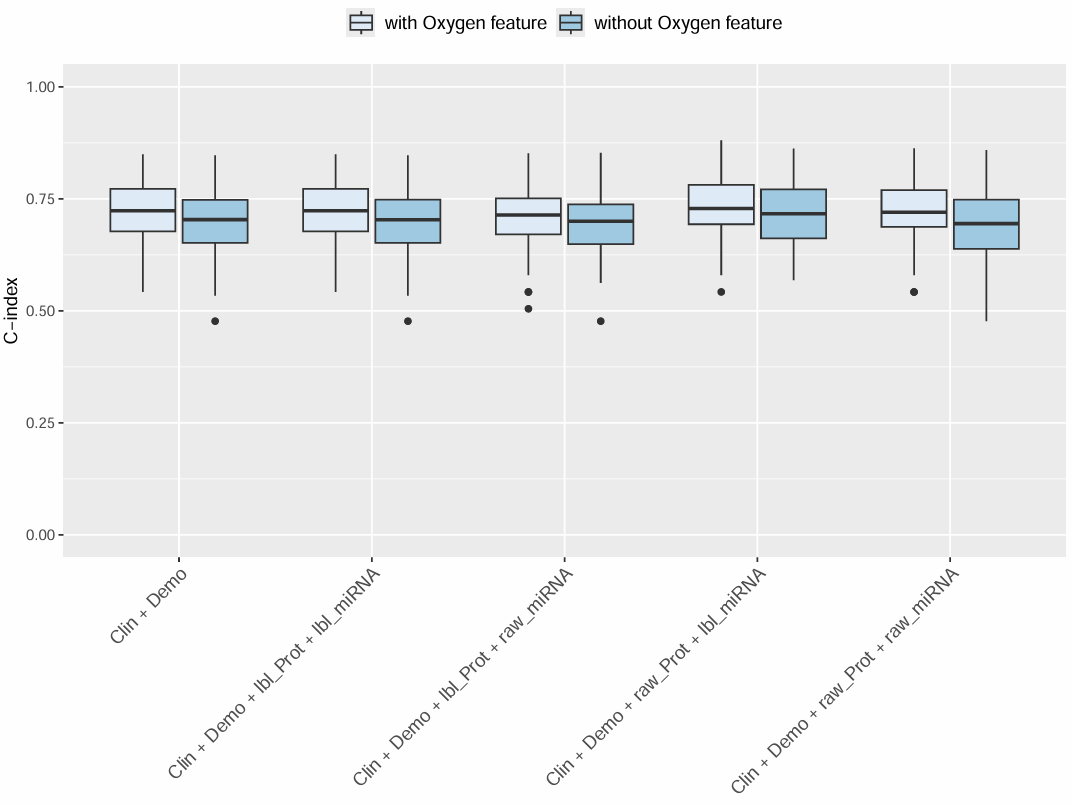


**Fig. S2** C-indices of models including and not including supplemental oxygen use at rest for the composite of time to death, lung transplant, or decline in forced vital capacity % predicted >10%.


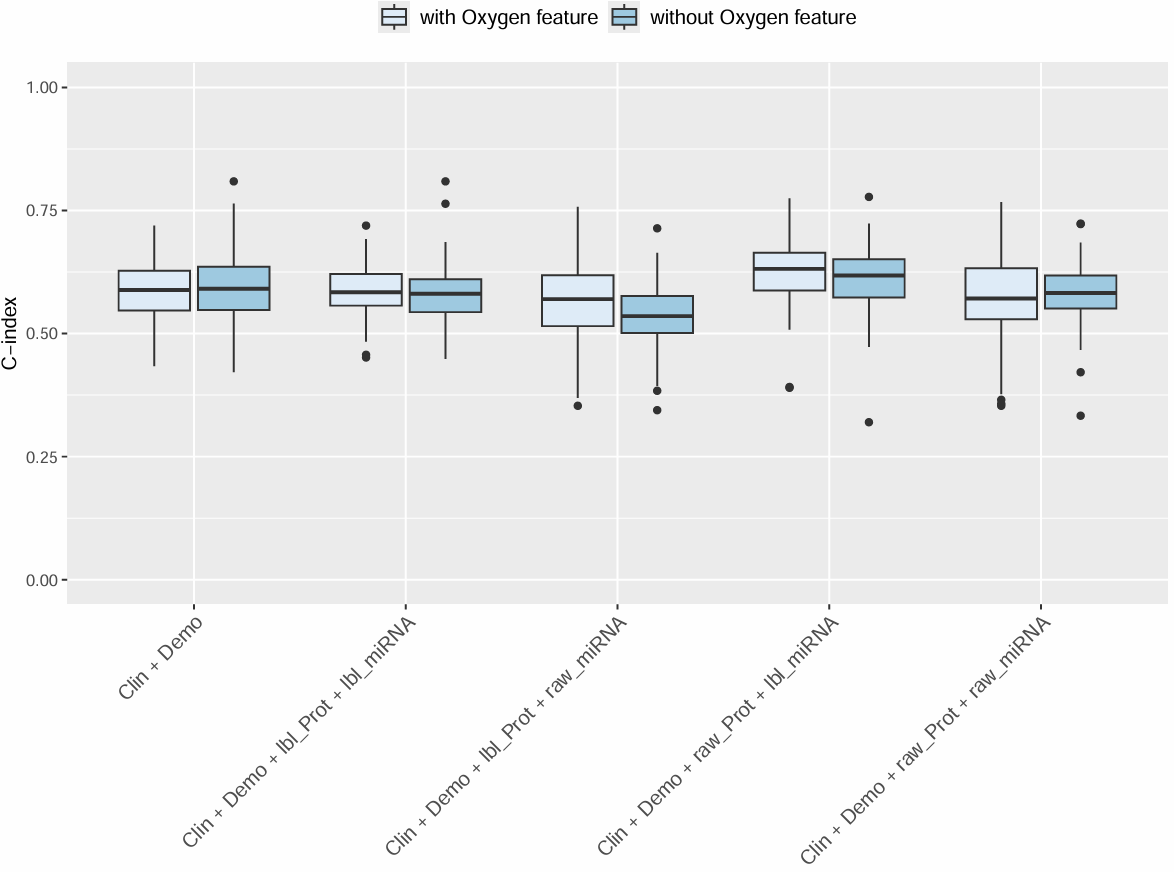


**Fig. S3** C-indices of models for the composite of time to death, lung transplant, or decline in diffusing capacity of the lungs for carbon monoxide (DLco) % predicted ≥15%.


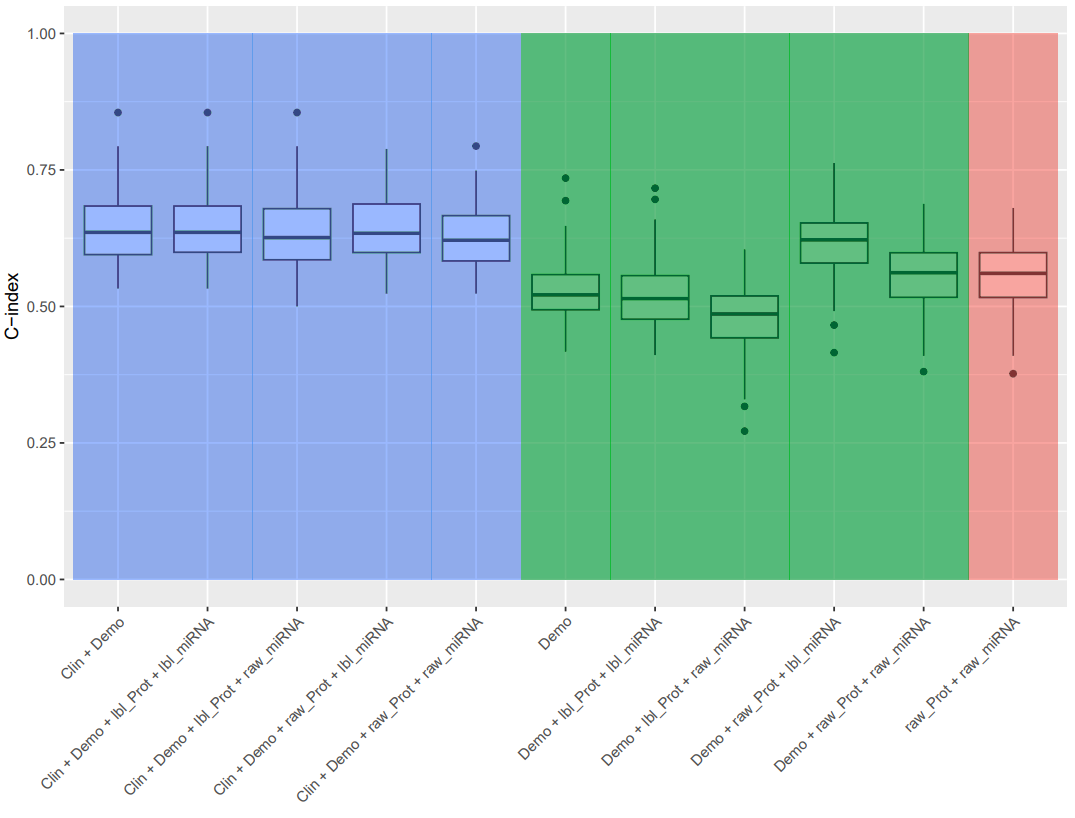


Blue shading (left panel) denotes models based on demographic and clinical characteristics with or without omics data. Green shading (middle panel) denotes models based on demographics with or without omics data. Red shading (right panel) denotes the model based on omics data alone.

Clin, clinical; demo, demographics; lbl_miRNA, cluster label of miRNA; lbl_prot, cluster label of protein; raw_miRNA, raw values of miRNAs; raw_prot, raw values of proteins.

**Fig. S4** C-indices of models for the composite of time to death, lung transplant, or respiratory hospitalization.


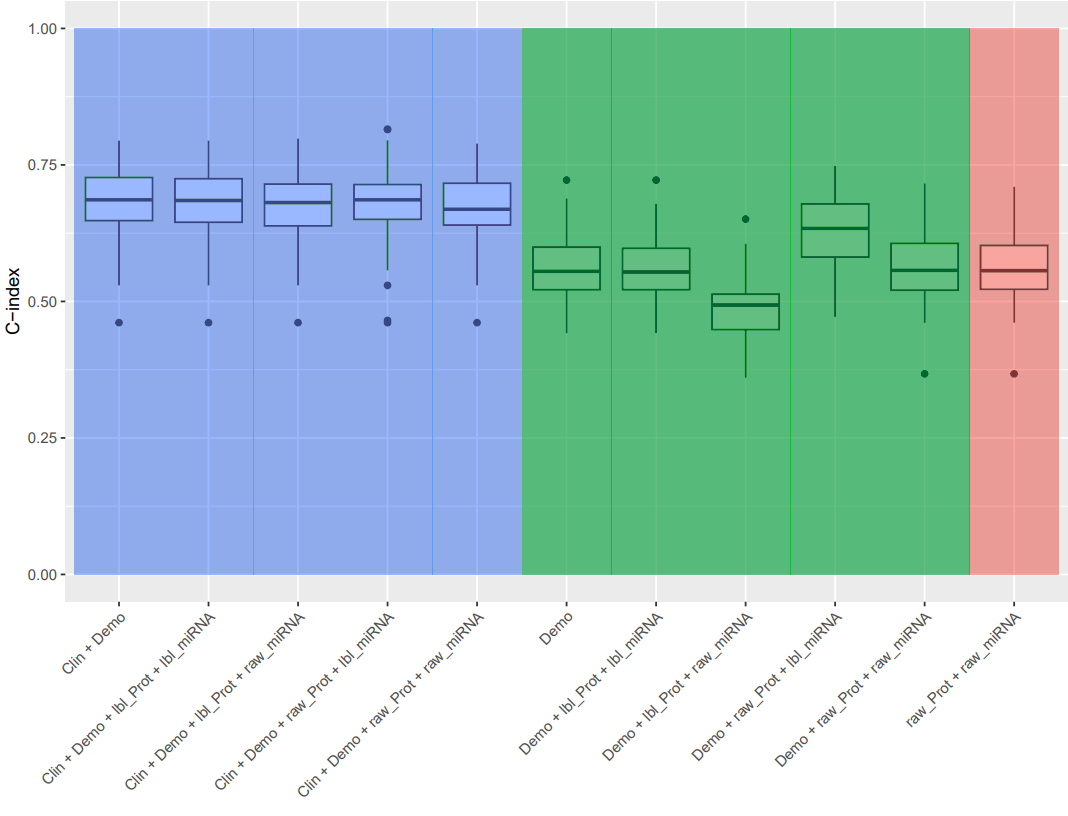


Blue shading (left panel) denotes models based on demographic and clinical characteristics with or without omics data. Green shading (middle panel) denotes models based on demographics with or without omics data. Red shading (right panel) denotes the model based on omics data alone.
Clin, clinical; demo, demographics; lbl_miRNA, cluster label of miRNA; lbl_prot, cluster label of protein; raw_miRNA, raw values of miRNAs; raw_prot, raw values of proteins.

**Fig. S5** Correlations between two clinical factors (forced vital capacity [FVC] and diffusing capacity of the lungs for carbon monoxide [DLco] % predicted) and 44 proteins.
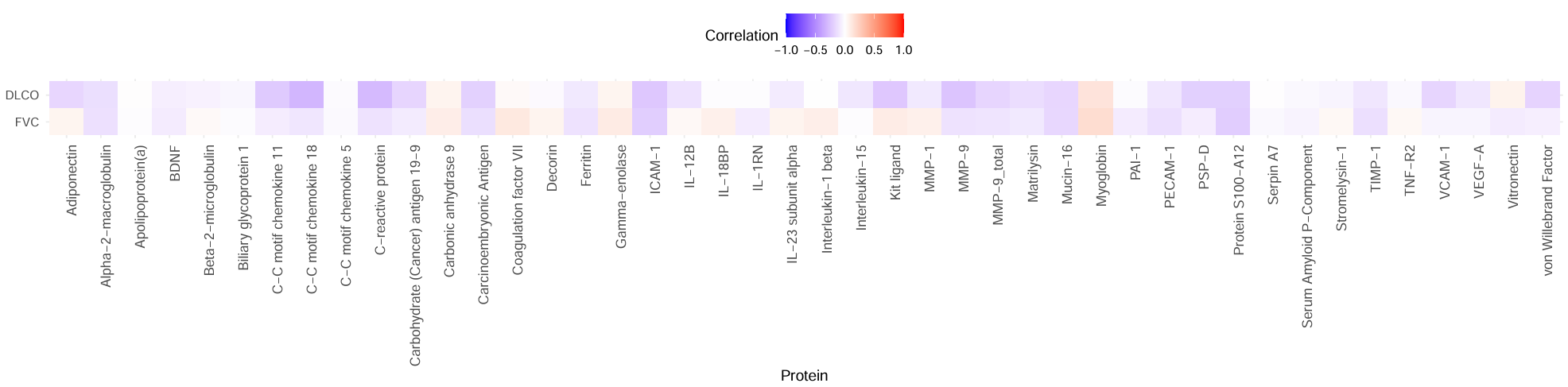


**Fig. S6** Variable importance of demographic, clinical and omics variables for composite of death, lung transplant, or decline in diffusing capacity of the lungs for carbon monoxide (DLco) % predicted ≥15%.


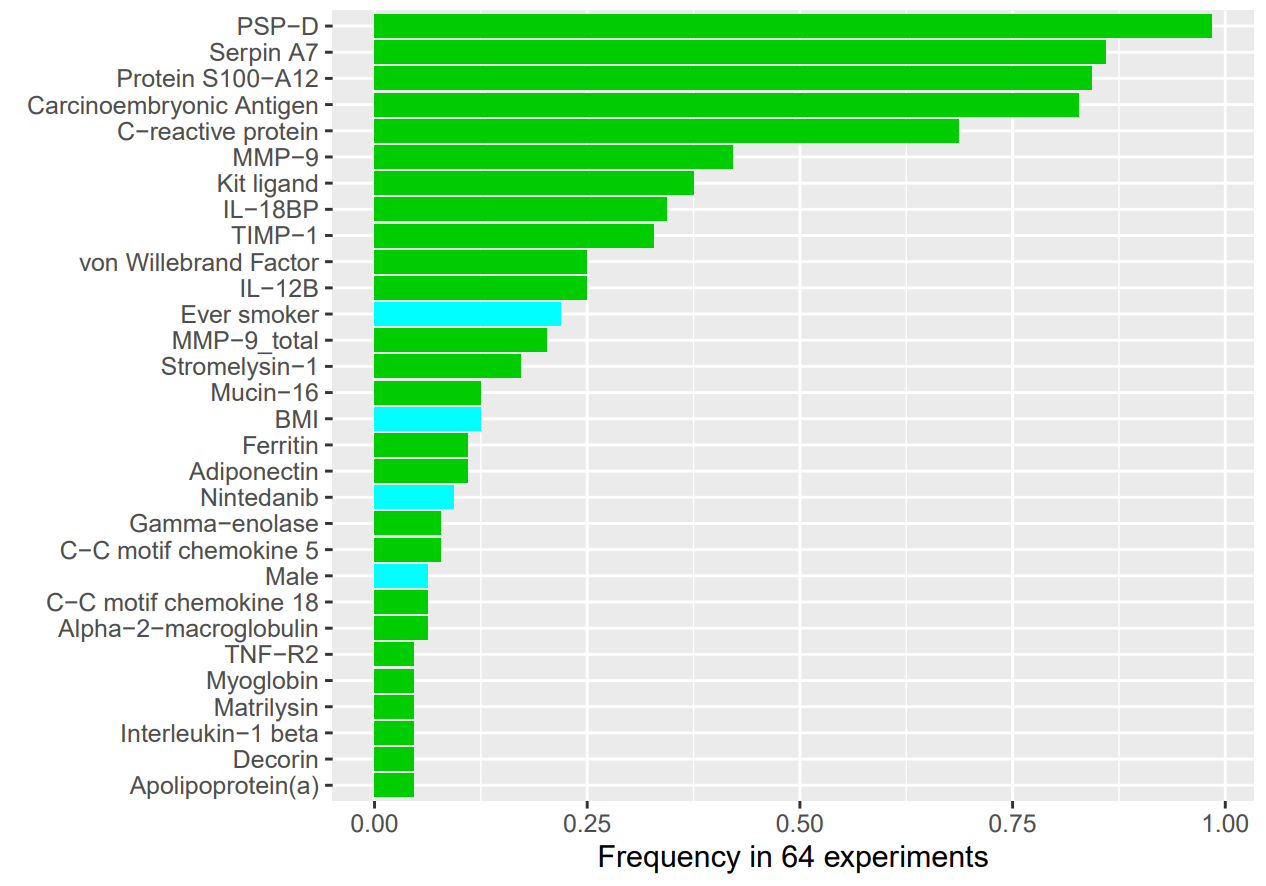


Green bars denote proteins and blue bars denote demographic variables.
Demo, demographics; lbl_miRNA, cluster label of miRNA; raw_prot, raw values of proteins.

**Fig. S7** Variable importance by selected frequency for composite of death, lung transplant, or respiratory hospitalization.


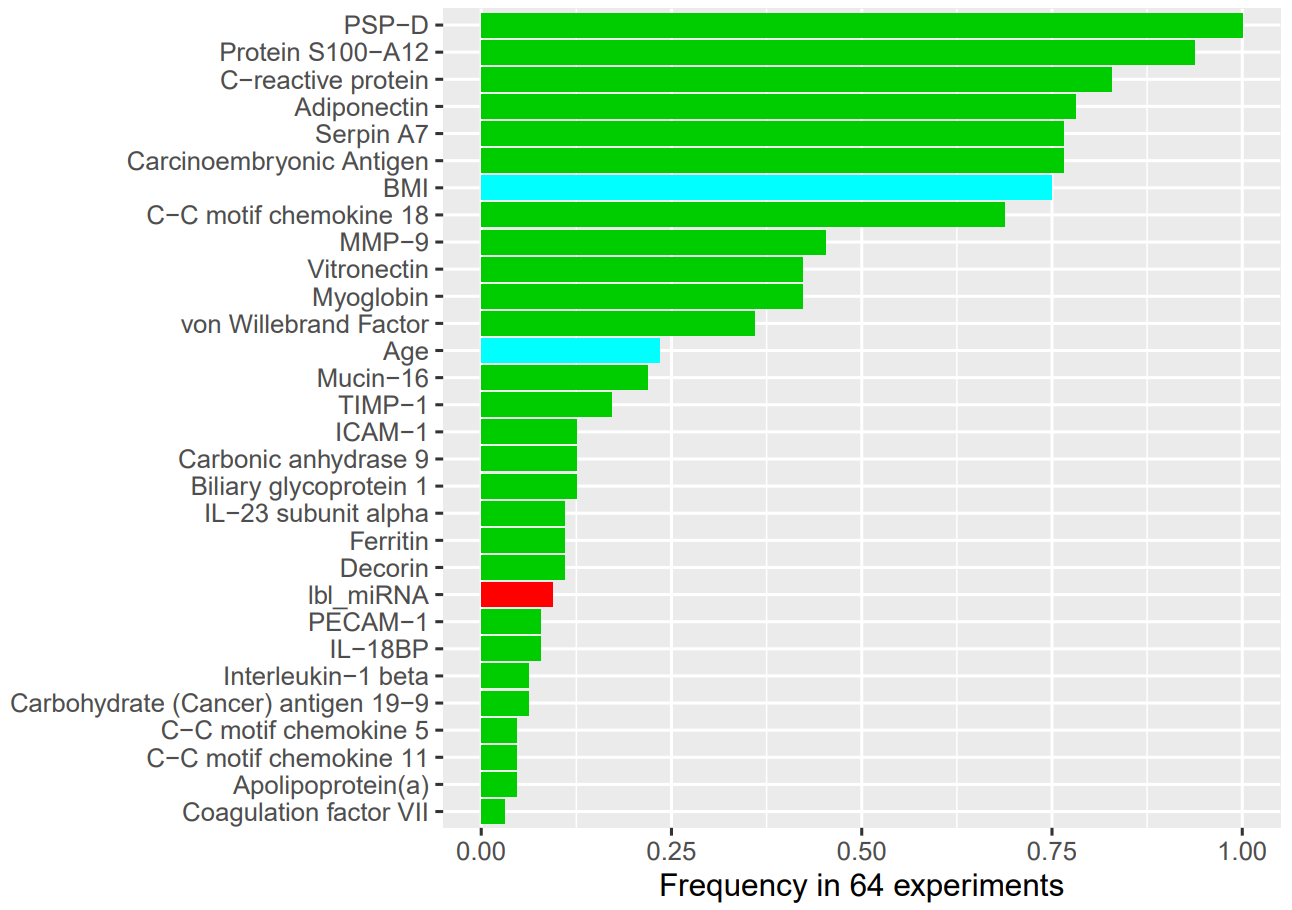


Green bars denote proteins, blue bars denote demographic variables and the red bar denotes the cluster label of miRNA.

Demo, demographics; lbl_miRNA, cluster label of miRNA; raw_prot, raw value of protein.

**Fig. S8** C-indices of refitted models using the top 4 proteins and other covariates alongside Lasso models for the composite of time to death or lung transplant. The left panel shows models based on demographic and clinical characteristics with or without omics data. The middle panel shows models based on demographics with or without omics data. The right panel shows the model based on omics data alone. Clin, clinical; demo, demographics; lbl_miRNA, cluster label of miRNA; lbl_prot, cluster label of protein; raw_miRNA, raw values of miRNAs; raw_prot, raw values of proteins.


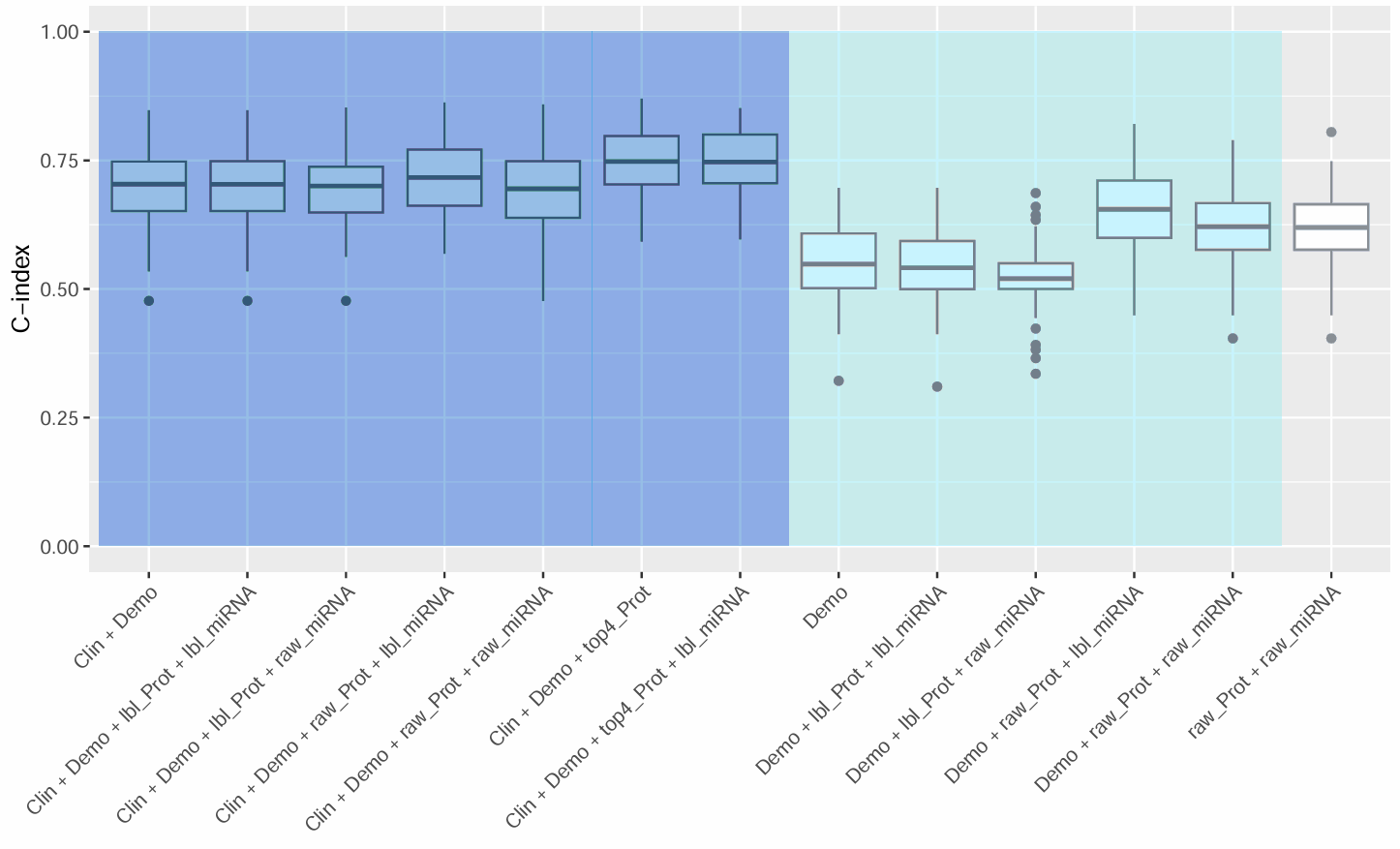


**Fig. S9** C-indices of refitted models with top 4 proteins and other covariates alongside Lasso models for the composite of time to death, lung transplant, or decline in FVC % predicted >10%. The left panel shows models based on demographic and clinical characteristics with or without omics data. The middle panel shows models based on demographics with or without omics data. The right panel shows the model based on omics data alone. Clin, clinical; demo, demographics; lbl_miRNA, cluster label of miRNA; lbl_prot, cluster label of protein; raw_miRNA, raw values of miRNAs; raw_prot, raw values of proteins.


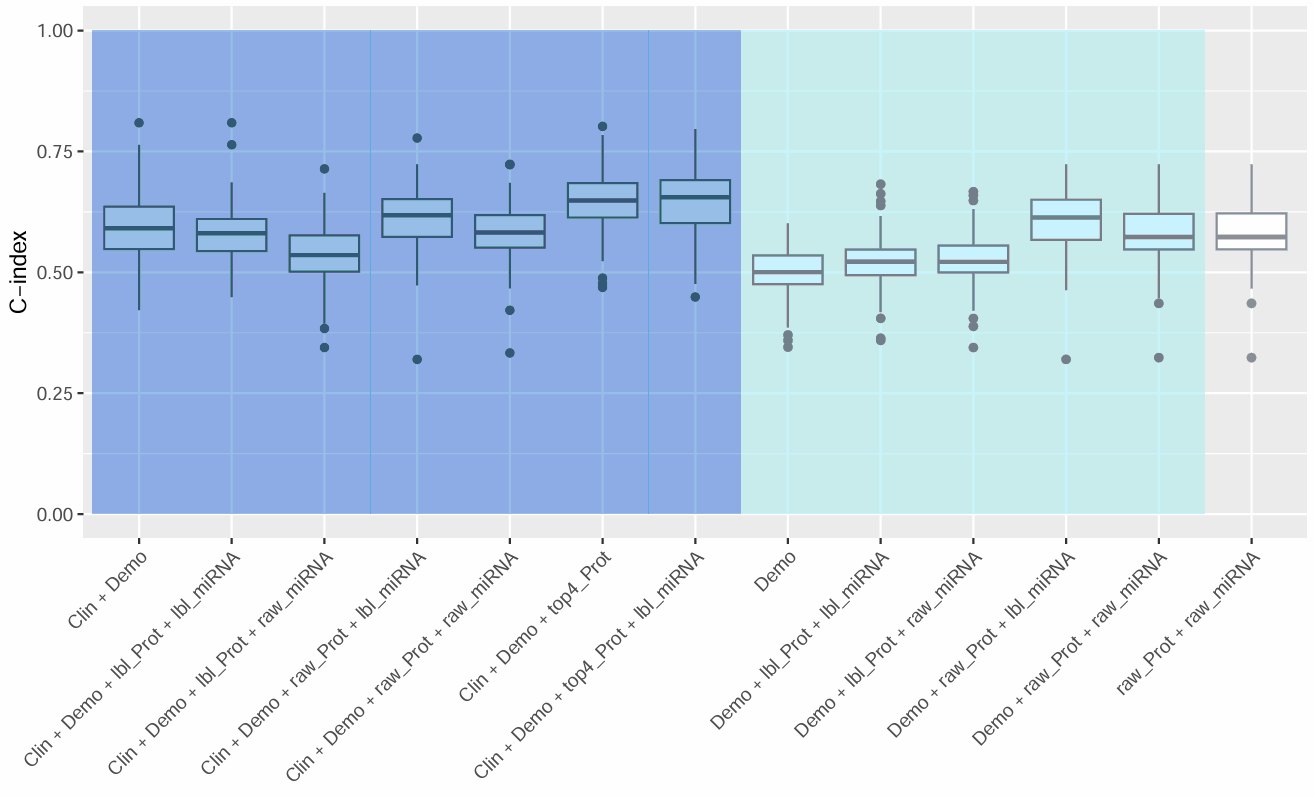


**Fig. S10** Area under the curve (AUC) of refitted models with top 4 proteins and other covariates alongside Lasso models for absolute decline in forced vital capacity (FVC) % predicted >10% at 1 year. The left panel shows models based on demographic and clinical characteristics with or without omics data. The middle panel shows models based on demographics with or without omics data. The right panel shows the models based on omics data alone. Clin, clinical; demo, demographics; lbl_miRNA, cluster label of miRNA; lbl_prot, cluster label of protein; raw_miRNA, raw values of miRNAs; raw_prot, raw values of proteins.


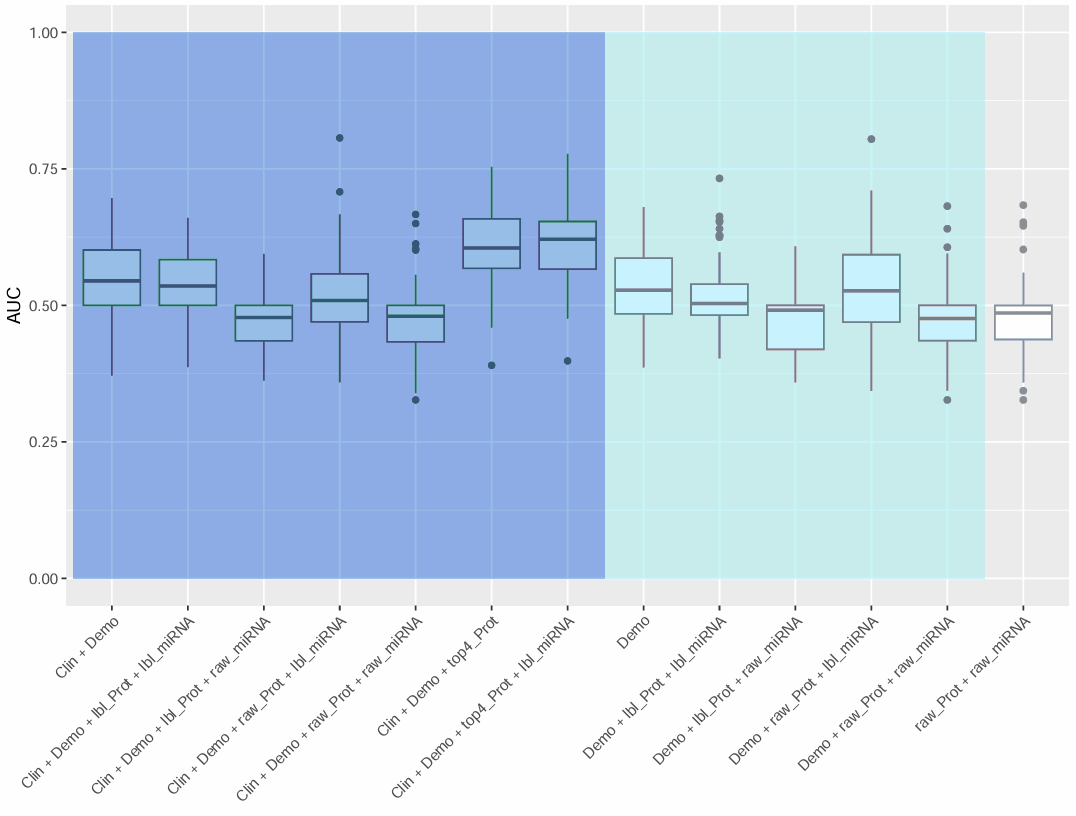


**Fig. S11** Area under the curve (AUC) of refitted models with top 4 proteins and other covariates alongside Lasso models for disease progression (decline in forced vital capacity [FVC] % predicted >10%, decline in diffusing capacity of the lungs for carbon monoxide [DLco] % predicted >15%, death, or lung transplant) at 1 year. The left panel shows models based on demographics and clinical characteristics with or without omics data. The middle panel shows models based on demographics with or without omics data. The right panel shows the models based on omics data alone. Clin, clinical; demo, demographics; lbl_miRNA, cluster label of miRNA; lbl_prot, cluster label of protein; raw_miRNA, raw values of miRNAs; raw_prot, raw values of proteins.


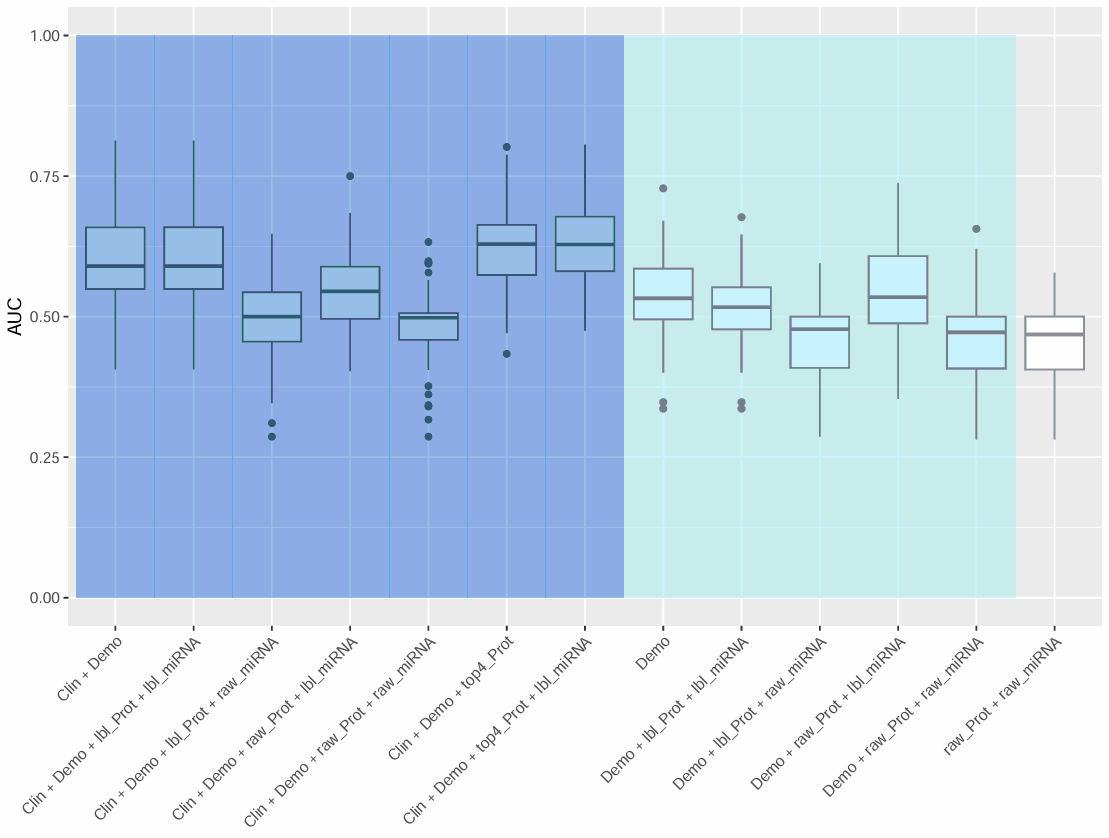

Supplement: Supplementary file 1 — Supplementary Material 1 [file 12931_2024_3015_MOESM1_ESM.docx]
